# Characterisation of tau in the human and rodent enteric nervous system under physiological conditions and in tauopathy

**DOI:** 10.1186/s40478-018-0568-3

**Published:** 2018-07-23

**Authors:** Arthur Lionnet, Matthew A. Wade, Anne-Gaëlle Corbillé, Alice Prigent, Sébastien Paillusson, Maddalena Tasselli, Jacques Gonzales, Emilie Durieu, Malvyne Rolli-Derkinderen, Emmanuel Coron, Emilie Duchalais, Michel Neunlist, Michael S. Perkinton, Diane P. Hanger, Wendy Noble, Pascal Derkinderen

**Affiliations:** 1 Inserm, U1235, 1 rue Gaston Veil, F-44035 Nantes, France; 2 Department of Neurology, CHU Nantes, F-44093 Nantes, France; 3 University Nantes, F-44000 Nantes, France; 4 King’s College London, Institute of Psychiatry, Psychology and Neuroscience, Department of Basic and Clinical Neuroscience, Maurice Wohl Clinical Neuroscience Institute, Rm 1.23, 5 Cutcombe Road, Camberwell, London, SE5 9RX UK; 5 Neuroscience, IMED Biotech Unit, AstraZeneca, Cambridge, CB21 6GH UK

**Keywords:** Tau, Tau isoform, Tau phosphorylation, Enteric nervous system, Progressive supranuclear palsy, Parkinson’s disease, Gut, Biopsy, Htau mouse

## Abstract

Tau is normally a highly soluble phosphoprotein found predominantly in neurons. Six different isoforms of tau are expressed in the adult human CNS. Under pathological conditions, phosphorylated tau aggregates are a defining feature of neurodegenerative disorders called tauopathies. Recent findings have suggested a potential role of the gut-brain axis in CNS homeostasis, and therefore we set out to examine the isoform profile and phosphorylation state of tau in the enteric nervous system (ENS) under physiological conditions and in tauopathies. Surgical specimens of human colon from controls, Parkinson’s disease (PD) and progressive supranuclear palsy (PSP) patients were analyzed by Western Blot and immunohistochemistry using a panel of anti-tau antibodies. We found that adult human ENS primarily expresses two tau isoforms, localized in the cell bodies and neuronal processes. We did not observe any difference in the enteric tau isoform profile and phosphorylation state between PSP, PD and control subjects. The htau mouse model of tauopathy also expressed two main isoforms of human tau in the ENS, and there were no apparent differences in ENS tau localization or phosphorylation between wild-type and htau mice. Tau in both human and mouse ENS was found to be phosphorylated but poorly susceptible to dephosphorylation with lambda phosphatase. To investigate ENS tau phosphorylation further, primary cultures from rat enteric neurons, which express four isoforms of tau, were pharmacologically manipulated to show that ENS tau phosphorylation state can be regulated, at least in vitro. Our study is the first to characterize tau in the rodent and human ENS. As a whole, our findings provide a basis to unravel the functions of tau in the ENS and to further investigate the possibility of pathological changes in enteric neuropathies and tauopathies.

**Electronic supplementary material:**

The online version of this article (10.1186/s40478-018-0568-3) contains supplementary material, which is available to authorized users.

## Introduction

The microtubule-associated protein tau is found predominantly in neurons, where it exists as a highly soluble protein that interacts with the cytoskeleton [25, 28]. Six different isoforms of tau are expressed in the adult human CNS via alternative splicing of the *MAPT* gene, which comprises 16 exons. Regulated inclusion of exons 2 and 3 yields tau isoforms with 0, 1, or 2 N-terminal inserts (0 N, 1 N, 2 N, respectively), whereas exclusion or inclusion of exon 10 leads to expression of tau isoforms with three (3R) or four (4R) microtubule-binding repeats [28]. The various splice combinations of tau are thus abbreviated 0N3R, 0N4R, 1N3R, 1N4R, 2N3R, 2N4R, encoding six protein isoforms ranging from 352 to 441 amino acids in length [25]. The function of tau is strongly affected by its phosphorylation status, which influences its ability to interact with microtubules and various signaling proteins [20, 57], as well as its localization and association with membranes [56, 63]. Under pathological conditions, aberrant assembly of highly phosphorylated tau into insoluble aggregates is observed in a range of neurodegenerative disorders, collectively referred to as tauopathies. Tauopathies encompass more than 20 clinicopathological entities, including Alzheimer’s disease (AD), progressive supranuclear palsy (PSP), Pick’s disease, all of which can be biochemically subclassified according to the predominance of tau isoforms found in the intracellular aggregates [43]. Tau aggregates found in tauopathies generally contain tau in an elevated state of phosphorylation [7, 29, 34] that is often aberrantly cleaved [31, 51]. Highly phosphorylated forms of tau are also found in other neurodegenerative diseases, including Parkinson’s disease (PD), where it often colocalises with abnormal alpha-synuclein [39, 66].

The enteric nervous system (ENS) is an integrated neuronal network distributed from the lower esophagus to the rectum*.* Compared to other components of the peripheral nervous system, the ENS shows some unique features that closely resemble the CNS and is sometimes referred to as ‘the brain-in-the-gut’ or the ‘second brain’. This close homology between the CNS and ENS suggests that a disease process affecting the CNS could also involve its enteric counterpart, as has already been described in variant Creutzfeldt-Jakob disease [33, 41] and PD [6, 21, 65]. Whether such a scenario can be extended to other neurodegenerative disorders such as tauopathies remains to be demonstrated, and this was one focus of the current study.

A few studies have shown that tau is expressed in rodent [30] and human [8, 17, 61] gastrointestinal (GI) tract, but no data are available about the distribution and phosphorylation pattern of tau isoforms in the ENS. Here, we examined the expression levels of tau isoforms, their phosphorylation profile and truncation in sigmoid colon biopsy specimens from PSP patients and compared them to samples from PD patients and controls. We examined the same tau characteristics in a mouse model of tauopathy in comparison to wild-type mice. Our results show the expression of two main human tau isoforms in the ENS. ENS tau is phosphorylated but is remarkably resistant to dephosphorylation with lambda phosphatase. We then examined the isoform profile and phosphorylation state of tau under physiological conditions in rat primary enteric neuron cultures, which showed that ENS tau phosphorylation can be modified, at least in vitro. These data provide the first detailed characterization of ENS tau in humans and rodents in health and tauopathies. Further investigation of tau modifications in the ENS in disease may provide valuable information about tau modifications that promote or prevent tau abnormalities spreading between the gut and brain in neurodegenerative diseases.

## Material and methods

### Human tissues

Samples of frozen temporal cortex from one post-mortem human brain devoid of neurodegeneration were obtained from the Neuropathology Department of Angers (Dr Franck Letournel) to serve as a control for the following experiments. Specimens of human colon were obtained from three neurologically unimpaired subjects who underwent colon resection for colorectal cancer. For all three tissues specimens, sampling was performed in macroscopically normal segments of uninvolved resection margins. Colonic sections were separated into muscle and submucosal/mucosal layer [36], which contain the myenteric and submucosal plexus respectively. Two out of three samples were frozen and kept at − 80 °C until further analysis by Western blot. The remaining sample was analyzed by immunohistochemistry.

Routine sigmoid colon biopsies were obtained during sigmoidoscopy/colonoscopy from 24 subjects, 10 with PD, 5 with PSP and 9 controls. All patients were recruited from the movement disorder clinic at Nantes University Hospital, France. Diagnosis of PD was made according to criteria provided by the United Kingdom Parkinson’s Disease Survey Brain Bank. PSP patients fulfilled the diagnostic criteria for possible or probable PSP. Control subjects were healthy subjects who had a routine colonoscopy performed for colorectal cancer screening. All controls subjects underwent a detailed neurological examination to rule out PD symptoms and cognitive deficiency. Except for control subjects 183 and 208 (Table 1) who had 6 biopsies, 4 biopsies per patient were taken during the endoscopic procedure. Biopsies were stored at − 80 °C until required.

**Table 1:** Demographics and characteristics of controls subjects and patients

Patient #	Age/sex	Diagnosis	DD
183	49/F	Control	–
188	67/F	Control	–
189	63/F	Control	–
190	45/M	Control	–
191	19/F	Control	–
208	76/M	Control	–
210	63/F	Control	–
214	69/F	Control	–
227	56/F	Control	–
162	56/F	PD	12
166	64/F	PD	11
167	67/M	PD	10
168	55/F	PD	4
171	71/M	PD	3
173	67/M	PD	11
175	70/M	PD	12
177	70/F	PD	8
178	53/F	PD	1
179	52/F	PD	4
170	63/F	PSP	4
176	72/M	PSP	4
185	72/F	PSP	11
187	75/M	PSP	5
228	76/F	PSP	1

Patient ID, age, sex, diagnosis of PD or PSP (including probable PSP) are shown in addition to disease duration (DD) in years

The sampling of human brain and colon was approved by the *Fédération des biothèques* of the University Hospital of Nantes, according to the guidelines of the French Ethics Committee for Research on Humans and registered under the no. DC-2008-402. Regarding sigmoid biopsies sampling, the study protocol was approved by the local Committee on Ethics and Human Research (*Comité de Protection des Personnes Ouest VI*), and registered on ClinicalTrials.gov (EnteroLark and ColoBioParker, identifier NCT01618383 and NCT01353183, respectively). Written informed consent was obtained from each patient and from each normal volunteer.

### Mouse tissues

Htau mice (B6.Cg-Mapt^tm1(EGFP)Klt^Tg(MAPT)8cPdav/J) were originally purchased from the Jackson laboratory (Bar Harbor, ME, USA) and maintained at King’s College London. Wild-type and tau knockout offspring of an identical background strain (C57Bl/6 J) were obtained via breeding. All housing and experimental procedures were carried out in compliance with the local ethical review panel of King’s College London under a UK Home Office project license held in accordance with the Animals (Scientific Procedures) Act 1986 and the European Directive 2010/63/EU. Two-month old male and female mice were used in this study. Animals were housed at 19–22 °C, humidity 55%, 12 h:12 h light: dark cycle with lights on at 07:30. Animals were culled using Schedule 1 methods, brains removed and snap-frozen on dry-ice. Sections of colon tissue were removed, with tissue from the distal portion of each part being cleaned and snap-frozen on dry-ice, prior to storage at − 80 °C for RNA extraction or biochemical analysis. The proximal portion from each part of colon along with the duodenum, jejunum and ileum were dissected with fine forceps to reveal the myenteric plexus as described previously [62].

### Rat tissues

Sciatic nerve sections were taken from two pregnant Sprague-Dawley rats (used for the generation of primary culture of rat ENS, see below) to serve as a positive control for big tau experiments [60].

### Primary cultures of rat ENS

Primary culture of rat ENS were generated using pregnant Sprague–Dawley rats (Janvier Laboratories SA, Le Genest-St-Isle, France) as previously described [11]. All housing and experimental procedures were carried out in compliance with the local ethical review panel of INSERM (agreement E. 44,011; INSERM, Nantes, France). Pregnant rats were killed by an overdose of CO_2_ followed by severing the carotid arteries. The small intestines of rat embryos were removed, diced in Hank’s Buffered Salt Solution (Sigma, Saint-Quentin Fallavier, France) and collected in 5 mL of Dulbecco’s modified Eagle’s medium (DMEM)-F12 (Gibco®, Life Technologies, Villebon sur Yvette, France) (1:1) for digestion at 37 °C for 15 min in 0.1% (*v*/v) trypsin (Sigma). The trypsin reaction was stopped by adding medium containing 10% fetal calf serum and then treatment with DNase I 0.01% (*v*/v) (Sigma) for 10 min at 37 °C. After triturating with a 10 mL pipette, cells were centrifuged at 750 rpm for 10 min. Cells were counted and then seeded at a density of 2.4 × 10^5^ cells/cm^2^ on 24-well plates previously coated with a solution of 0.5% (v/v) gelatin in sterile phosphate buffered saline. After 24 h, the medium was replaced with a serum-free medium DMEM-F12 (1:1) containing 1% (v/v) of N-2 supplement (Life Technologies). Cultures were maintained for 14 days.

### Treatment of rat ENS primary cultures with serine/threonine phosphatases inhibitors

After 14 days in vitro (DIV), cells were treated with a cocktail of three phosphatase inhibitors including 1 μM okadaic acid, 1 μM ciclosporine A and 6.75 μM sanguinarine (Sigma) for broad-spectrum inhibition of serine/threonine phosphatases, or with vehicle (DMSO, Sigma) for one hour.

### Dephosphorylation of tissues and cell lysates

For dephosphorylation experiments, cells or tissues were homogenised in a buffer containing 100 mM NaCl and 50 mM Tris-Cl at pH 7.4 with 1% (v/v) IGEPAL® CA-630 and a protease inhibitors cocktail without EDTA (Roche, Neuilly sur Seine, France) using either a “Precellys 24” (Bertin technologies, St Quentin-en-Yvelines, France) or a Tissue Master 125 (Omni International, Kennesaw, GA, USA) tissue homogenizer and followed by sonication with “vibracell 75 186” device (Sonics, Newton CT, USA). Homogenates were centrifuged at 16,300 g for 20 min at 4 °C with an Eppendorf 5415R centrifuge (Eppendorf, Hamburg, Germany), sonicated for 10 s and protein amounts normalized following a BCA protein assay (ThermoFisher, Waltham, MA, USA). Samples were diluted to 1.0 mg/mL protein using homogenisation buffer and incubated with 20 U/μL lambda phosphatase in MnCl_2_ and enzyme buffer as supplied with the lambda protein phosphatase kit (New England Biolabs, Ipswich, MA, USA) for 3 h at 30 °C. The reaction was stopped by the addition of sample buffer (National Diagnostics, Hull, UK or Life Technologies, Courtaboeuf, France) and heating to 95 °C for 5 min. Control samples were treated identically without the addition of lambda phosphatase.

### SDS-PAGE and western blot

For dephosphorylation experiments, cells or tissues were processed as described above. For experiments that did not require dephosphorylation, cells or tissues were lysed in RIPA lysis buffer (Merck Millipore, Fontenay sous Bois, France). Western blots were performed as we previously described [10] using NuPAGE™ 10% Bis-Tris Protein Gels (Life Technologies, Courtaboeuf, France). The primary anti-tau antibodies used are listed in Table 2. Phospho-ERK (Cell signaling, Ozyme, France 1:2000 dilution) and PGP 9.5 antibodies (Abcam, France, 1:1000 dilution) were used for the evaluation of phosphatase treatment and as loading control, respectively.

**Table 2:** Tau antibodies used in this study

Name	Specificity	Epitope (a.a)	Source and dilution
A0024 Tau	All tau isoforms	243–441 (2N4R)	Dako, rp (WB 1:1000; IHC 1:500)
TAU-5	All tau isoforms	210–241 (2N4R)	ThermoFisher, mm (WB 1:1000)
Tau-1	All tau isoforms	189–207 (2N4R)	Merck, mm, clone PC1C6 (WB 1:2000)
TP70	All tau isoforms	428–441 (2N4R)	IOP, KCL, rp (WB 1:500)
Anti tau RD3	3R tau Isoforms	267–282 (2N3R)	Merck, mm, clone 8E6 (WB 1:1000; IHC 1:500)
Anti tau RD4	4R tau isoforms	275–291 (2N4R)	Merck, mm, clone 1E1/A6 (WB 1:1000)
Anti 4R-tau	4R tau isoforms	NS	Cosmo bio co., rp (WB 1:2000; IHC 1:1000)
Anti 0 N-tau	0 N Tau isoforms	39–50 (0N3R)	BioLegend, mm (WB 1:500)
AT8	Tau ℗ S202/T205	Tau ℗ S202/T205	Innogenetics, mm (WB 1:1000)
PHF-1	Tau ℗ S396/S404	Tau ℗ S396/S404	Gift from Peter Davies, mm (WB 1:500)
PHF13	Tau ℗ S396	Tau ℗ S396	Cell Signaling, mm (WB 1:1000)
CP13	Tau ℗ S202	Tau ℗ S202	Gift from Peter Davies, mm (IHC 1:200)

The name, specificity, epitope, source and dilution of the antibodies used in this study are shown.

*Abbreviations*: *a.a.* amino-acids, *IHC* immunohistochemistry, *IOP, KCL* Institute of Psychiatry, King’s college London, *mm* mouse monoclonal, *NS* not specified, *rp* rabbit polyclonal, *WB* western blot

### Immunohistochemistry

For mouse GI tract tissues, following the excision of myenteric plexus from mouse colon, tissue segments were incubated in combined blocking (50 mM tris-buffered saline [TBS] pH 7.4 containing 5% bovine serum albumen [BSA] and 0.05% tween-20) and permeabilisation (50 mM TBS pH 7.4, 0.1% triton X-100) solutions overnight at 4 °C. Primary antibodies (Table 2) in blocking solution were incubated with gut tissues overnight at 4 °C. Following washing in 50 mM TBS the appropriate fluorescently-tagged secondary antibody was added for 3 h at ambient temperature, the antibodies removed by washing and Hoechst 33258 added for 3 min. Images were acquired using a CTR5000 digital camera (Leica Microsystems, Cambridge, UK) attached to a Leica DM5000B fluorescence microscope with Leica AIF lite software.

For human tissues, fixed human tissues were embedded in paraffin using an embedding station (LEICA EG1150C) and sections (3 μm) were cut using a microtome (LEICA RM2255). The sections were deparaffinised by bathing twice in xylene (for 5 min each) and taken through graded concentrations of ethanol (100, 95, 70, 70%, respectively for 3 min each). After a rinse in distilled water, slides were washed in PBS and antigen retrieval was performed using a sodium citrate solution (2.94 g Sodium Citrate Tribase; 1 L ultrapure water; 500 μL Tween 20; pH 6) at 95 °C for 20 min. Slides were incubated in NH_4_Cl (100 mM) for 15 min before incubation in PBS-0.5% triton X-100 for 1 h and blocking for 2 h in 10% horse serum in PBS-0.5% triton X-100. Primary antibodies (Table 2) were incubated overnight at 4 °C, and following washing, secondary antibodies were added for 2 h at room temperature. Images were acquired with an Olympus IX 50 fluorescence microscope coupled to a digital camera (model DP71, Olympus).

### RNA extraction and RT-PCR

Frozen proximal colon and cortex from htau, wild-type and tau knockout mice was homogenised in approximately 100 mg/mL Quiazol® supplied with the Quiagen RNA LipidEasy kit (Qiagen, Hilden, Germany), and RNA was extracted following the manufacturer’s protocol. The RNA obtained was eluted in ultrapure H_2_O and its concentration and purity determined using a NanoDrop spectrophotometer (Thermo Scientific, Waltham, MA, USA). Samples were diluted to 1 μg RNA/15 μL RNAse-free H_2_O, heat-shocked for 3 min at 72 °C to break down double-stranded structures and returned immediately to ice. One μg RNA per sample was reverse transcribed using a Superscript III reverse transcriptase assay kit (Life Technologies, Paisley, UK) according to the manufacturer’s instructions. The resulting cDNA was stored at − 20 °C until use. To examine the alternate splicing of the microtubule binding domain repeat region encoded by exon 10, primers were used that specifically recognize mouse or human exons 9 and 11 as described by Duff et al. [16]. Primer sequences were: mouse exon 9F 5’-CCCCCTAAGTCACCATCAGCTAGT, mouse exon 11R 5’-CACTTTGCTCAGGTCCACCGGC, human exon 9F 5’-CTCCAAAATCAGGGGATCGC, human exon 11R 5’-CCTTGCTCAGGTCAACTGGT. Splicing around the N terminal insert domain encoded by exons 2 and 3 was detected using primers that recognize exons 1 and 5. Primer sequences used were: mouse exon 1F 5’-TCCGCTGTCCTCTTCTGTC, mouse exon 5R 5′- TTCTCGTCATTTCCTGTCC, human exon 1F 5′- TGAACCAGGATGGCTGAGC, human exon 5R 5’-TTGTCATCGCTTCCAGTCC. Annealing temperatures were 64 °C (all *MAPT* primers), 62 °C (M1F/M5R) and 68 °C (M9F/M11R). 35 reaction cycles were used for all. Mouse and human-specific RT-PCR products were analysed by agarose gel electrophoresis. Products corresponding to exon 10+ tau mRNA (4R) are 390 base pairs (bp), while products corresponding to exon 10- mRNA (3R) are 297 bp. RT-PCR products containing tau mRNA with exons 2 and 3 (2 N) are 428 bp, 2 + 3- mRNA products (1 N) are 341 bp, and 2–3- mRNA products (0 N) are 253 bp.

### Statistics

All data shown are mean ± SEM. Statistical analyses was conducted using GraphPad software version 5.00 (San Diego California, USA). For comparisons of means between groups, Kruskal-Wallis tests were performed. Differences were deemed statistically significant when *p* < 0.05.

## Results

### The expression pattern of tau isoforms is different in adult human brain and gut

**Fig. 1:**
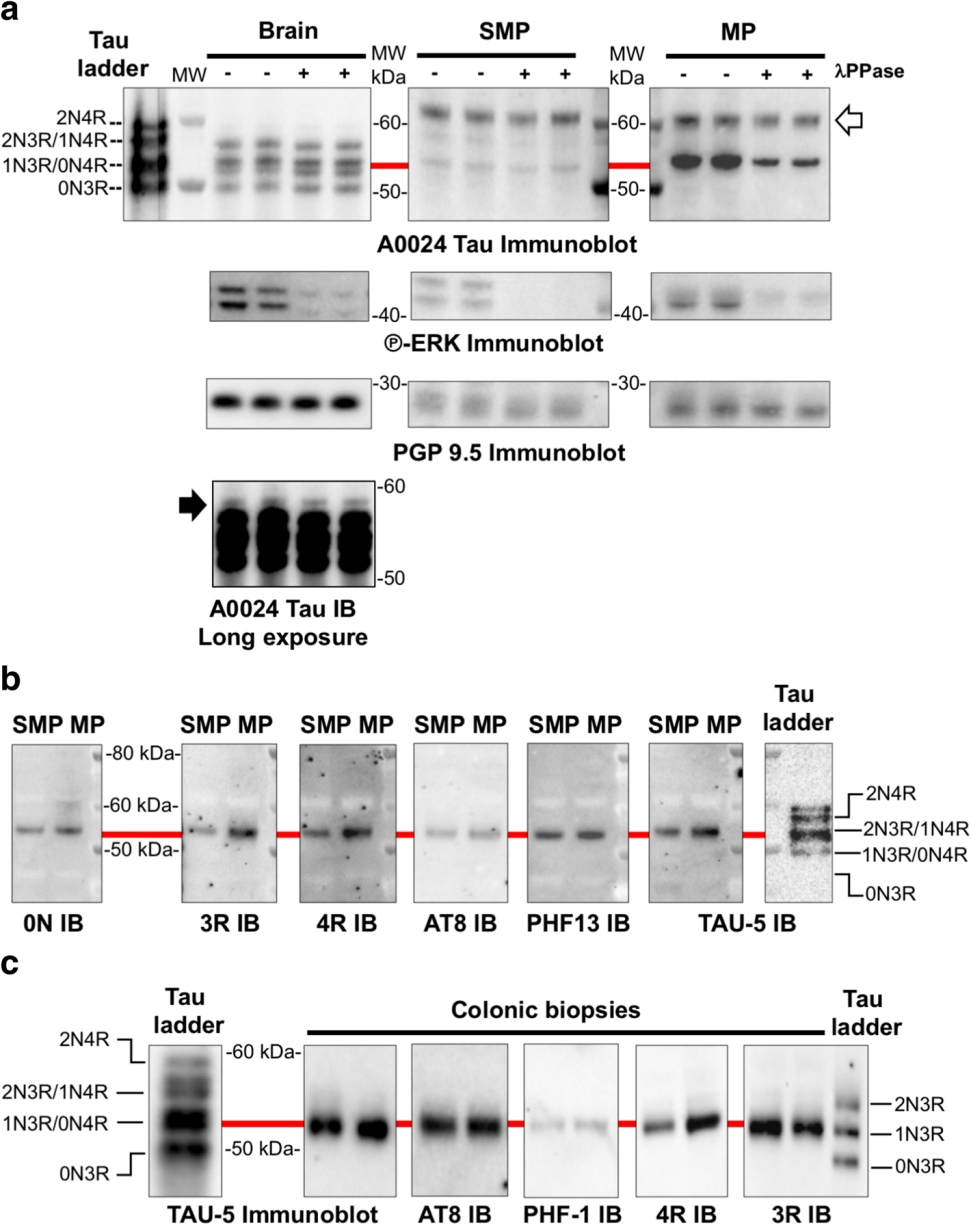
Tau isoforms and phosphorylation in adult human ENS. **a** Human brain and colon tissue lysates (submucosal and muscle layers, which contains the submucosal (SMP) and myenteric plexus (MP), respectively) were subjected to immunoblot analysis using the pan-Tau antibody A0024. Lysates were treated (+) or not (−) with lambda phosphatase before immunoblotting. The effectiveness of dephosphorylation was confirmed by phospho-ERK immunoblot (P-ERK immunoblot). Tau antibody A0024 detected all six tau isoforms in the recombinant human tau ladder and brain samples (the 2N4R was only visible on long exposure immunoblots, black arrow). The non-specific band detected by Tau antibody A0024 in the ENS is marked by a white arrow. An antibody against protein gene product (PGP) 9.5 was used as a loading control. **b** Colon tissue lysates (SMP and MP) were subjected to immunoblot analysis using antibodies specific to 0 N, 3R, 4R tau, the pan-tau TAU-5 antibody, and the phospho-specific tau antibodies AT8 (phos-Ser202/Thr205) and PHF13 (phos-Ser396). **c** Sigmoid colon biopsies lysates from 2 control subjects (#183 and 208, Table 2) were subjected to immunoblot analysis using the TAU-5 antibody, antibodies specific to the 3R and 4R tau isoforms and the phospho-specific tau antibodies AT8 and PHF-1. In all experiments, the banding pattern was compared to that of tau ladder which contains all six recombinant tau isoforms. The red line shows the comigration of the observed bands with 1 N3/0N4R. The results shown in (**a**), (**b**) and (**c**) are representative of 3, 2 and 5 independent experiments, respectively

**Fig. 2:**
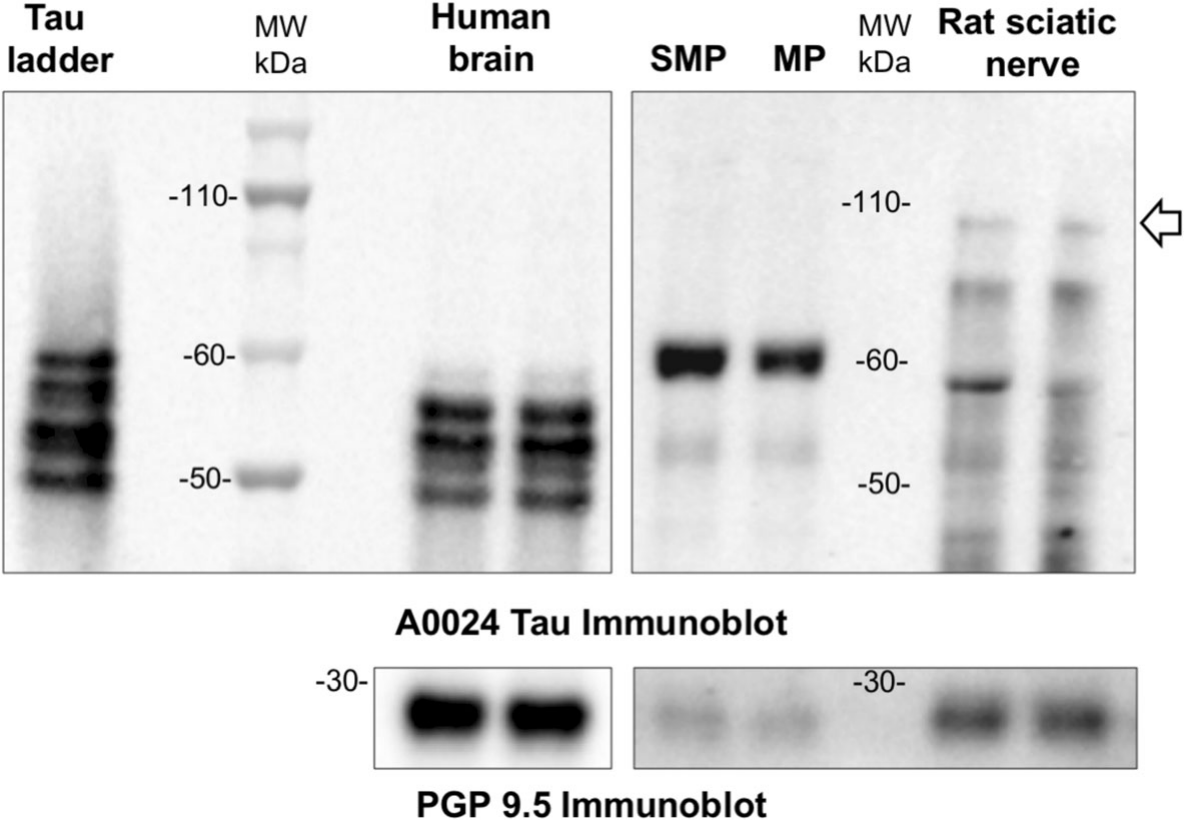
Big tau is not detected in adult human ENS. Human brain and colon tissue lysates (SMP and MP) were subjected to immunoblot analysis using the pan-Tau antibody A0024. Rat sciatic nerve lysates were used as positive control to detect big tau (white arrow). PGP 9.5 was used as a loading control. Images are representative of five independent experiments

In adult human brain, the six tau isoforms are phosphorylated resulting in reduced electrophoretic mobility on SDS-PAGE compared to recombinant tau [25]. In order to identify the tau isoforms expressed in the human ENS, colonic samples from healthy subjects treated or not with lambda phosphatase [35] were analyzed by western blot using the A0024 tau antibody that recognizes all six tau isoforms. ENS samples were compared to dephosphorylated and non-dephosphorylated brain samples as well as to a recombinant tau ladder. The banding pattern was markedly different between brain and colonic samples (Fig. 1a). The A0024 Tau antibody detected one major band migrating at 53–54 kDa in both the submucosal and muscle layers (which contain the submucosal and myenteric plexus, respectively and therefore are referred to as SMP and MP) (Fig. 1a). This band migrated only slightly faster after dephosphorylation of SMP and MP samples despite the efficiency of the dephosphorylation treatment being validated by phospho-ERK immunoblot, Fig. 1a). The major band detected in ENS samples comigrated with 0N4R-1N3R detected in human brain samples and the recombinant tau ladder (red line in Fig. 1a) and was also observed when a pan-tau (TAU-5) antibody was used (Fig. 1b). In addition, a fainter band around 57–58 kDa in SMP and a strong immunoreactive band at 62 kDa (white arrow) in both SMP and MP were also observed when the A0024 tau antibody was used (Fig. 1a). These two bands are most likely non-specific as they were not observed with TAU-5 (Fig. 1b) or with other specific antibodies subsequently used in this study (Figs. 1b, c and 2).

To further refine this analysis, we used 3 commercially available isoform-specific tau antibodies. Two of these antibodies directed against 3R and 0 N-tau have been shown to be highly specific in a recent comprehensive study that tested the specificity of tau antibodies using immunoblotting [19]. In addition, we used a 4R-tau antibody that only detects 4R tau isoforms in human brain lysates and in tau ladder (Additional file 1: Figure S1). All of these antibodies detected a single 53–54 kDa-band that comigrates with the major band detected by TAU-5 and with 0N4R-1N3R in the recombinant tau ladder (Fig. 1b).

Until recently, analysis of the ENS in humans was mainly performed using full thickness specimens of the gut obtained during surgery or autopsy. However, several recent studies have shown that the ENS is accessible and analyzable through routine GI biopsies, which can be processed to measure quantitative differences in neuronal and/or glial markers [5, 23, 44]. We therefore analyzed the expression levels of tau in routine sigmoid biopsies from 2 control subjects (#183 and 208, Table 1) with the pan-tau antibody TAU-5 and with the 3R and 4R isoform-specific antibodies. The immunoblotting pattern observed with these 3 antibodies in biopsies was similar to those observed in colonic SMP and MP samples (Fig. 1c).

“Big” or peripheral tau is a tau isoform specifically expressed in the peripheral nervous system, including trigeminal, dorsal root and sympathetic ganglia as well as sciatic nerve. It differs from the 2N4R tau isoform by a 254 amino-acid insert located in the amino-terminal half and migrates at 110 kDa on SDS/PAGE [27]. To determine whether big tau is expressed in the ENS, human colon tissue lysates were analyzed by Western blot using Tau A0024 antibody. Rat sciatic nerve lysates were used as positive controls [60]. Tau A0024 detected the expected low molecular weight tau isoforms between 45 and 60 kDa in human colon and rat sciatic nerve, however a 110 kDa migrating band was only observed with rat sciatic nerve lysates (Fig. 2).

When taken together, these results show that 1N3R and 0N4R are the two main tau isoforms that are expressed in human adult colon and these two isoforms can be detected in routine GI biopsies. In addition, our work indicates that big tau is not expressed in the adult human ENS.

### Tau isoforms are differentially expressed in the gut and brain of tauopathy mice

**Fig. 3:**
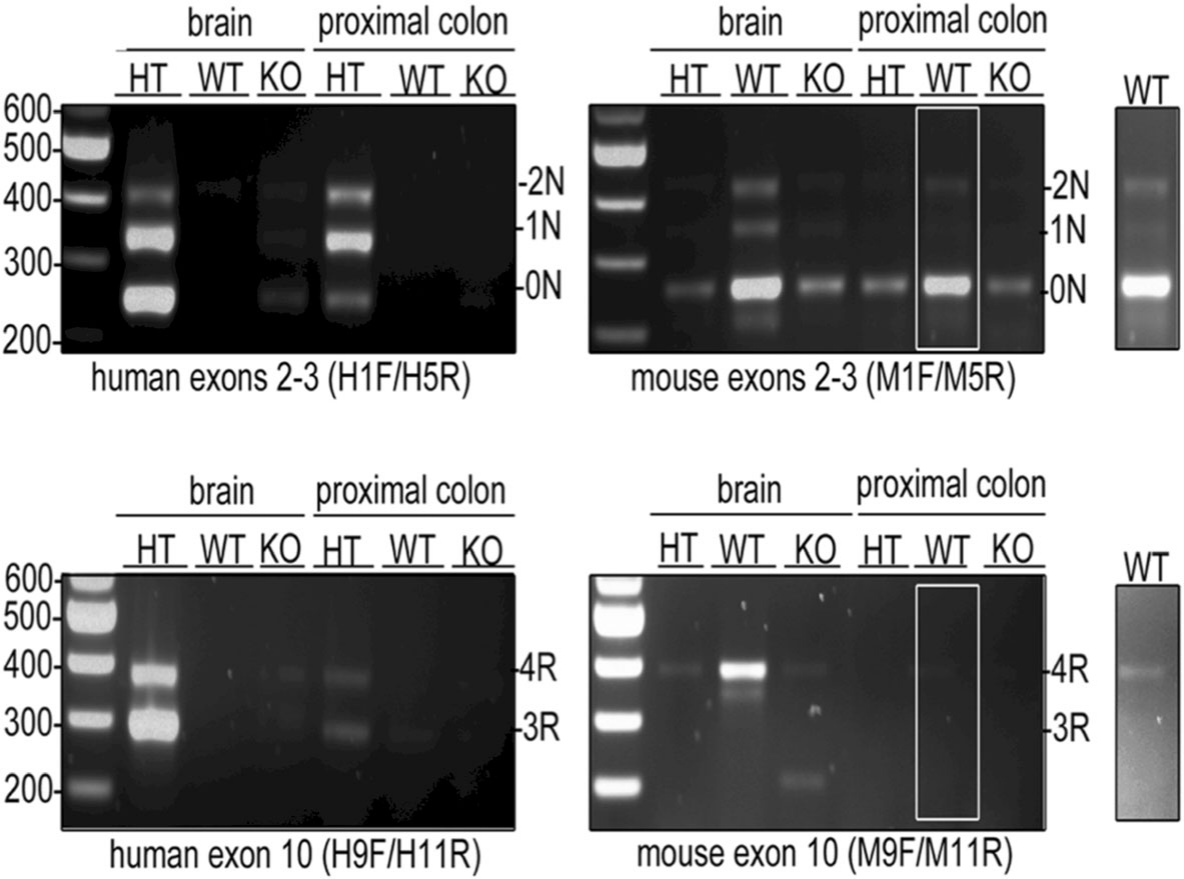
Detection of tau in htau and wild-type mouse brain and proximal colon. Htau (HT), wild-type (WT) and tau knock-out (KO) brain and proximal colon cDNA was amplified using PCR with human- and mouse-tau specific primers to detect the expression of exon 2–3- (0 N), 2 + 3- (1 N), 2 + 3+ (2 N), 10- (3R) and 10+ (4R) tau isoforms. Gel images show detection of human and mouse 0 N, 1 N and 2 N tau. An insert shows a higher intensity portion of image to illustrate tau products in proximal colon. Numbers correspond to base pairs of a DNA ladder. The expected position of PCR product is indicated to the right of each panel. *N* = 3

To determine if tau is also differentially expressed in the ENS and CNS of mice, we used the transgenic htau mouse model which expresses exclusively the six wild-type human isoforms of tau under the control of the *MAPT* promoter [3]. The enteric expression profile of tau isoforms in these transgenic mice was compared to that observed in wild-type mice. Tau knockout mice were examined as an additional control. To this end, RNA from 2-month-old, wild-type, tau knockout and htau mouse proximal colon was reverse transcribed to cDNA and amplified with PCR. Brain tissue from the same mice was used for comparison. Primers were designed, based on those previously described by Duff et al. [16], to detect splicing of human tau exons 2 and 3. This allowed amplification of products corresponding to 0 N, 1 N and 2 N human tau that were detected in htau, but not wild-type or tau knockout, brain and proximal colon (Fig. 3). Transcripts of 3R and 4R *MAPT* were also observed in htau brain and proximal colon when inclusion of exon 10 was assessed using primers specific to human tau exons 9 and 11 (Fig. 3). Thus, 0 N, 1 N, 2 N, 3R and 4R human tau transcripts are expressed in htau proximal colon.

Primers against mouse tau were also used to allow detection of 0 N, 1 N and 2 N transcripts in wild-type mouse brain and proximal colon (Fig. 3a). A weak non-specific PCR product corresponding to the predicted size of 0 N tau was also amplified in htau and tau knockout samples with these primers. In addition, 4R, but not 3R *Mapt* was detected in WT mouse brain, and a weak signal was also apparent in proximal colon (Fig. 3a). These transcripts were not amplified in htau or tau knockout tissues. Thus, wild-type mice express 0 N, 1 N, 2 N and mainly 4R tau in gut and brain.

The detection of multiple products in a single lane, each corresponding to a different tau isoform, allows each product to act as an internal control for the other transcripts. This allowed us to make comparisons between the relative abundance of tau isoforms in different tissues. Htau brain showed 0 *N* > 1 *N* > 2 N relative abundance of tau isoforms, in keeping with previous observations in adult mice [59]. Htau mouse brain also showed greater exclusion of tau exon 10 (3R > 4R), as previously reported [3] and in contrast to adult wild-type mice where mainly 4R tau is expressed (Fig. 3; [59]). Thus, htau brain mirrors human brain in that both 3R and 4R tau are expressed, albeit that under physiological conditions these isoforms are expressed in approximately equal proportions in the human CNS [4]. In contrast, in htau proximal colon, PCR transcripts showed an altered relative abundance of 1 N > 2 *N* > 0 N (Fig. 3), and there appeared to be approximately equal inclusion and exclusion of exon 10 (3R ≈ 4R). These data suggest differential expression of tau isoforms in the ENS of htau mice in comparison to those in brain.

### Tau protein is expressed throughout the human and mouse myenteric plexus

**Fig. 4:**
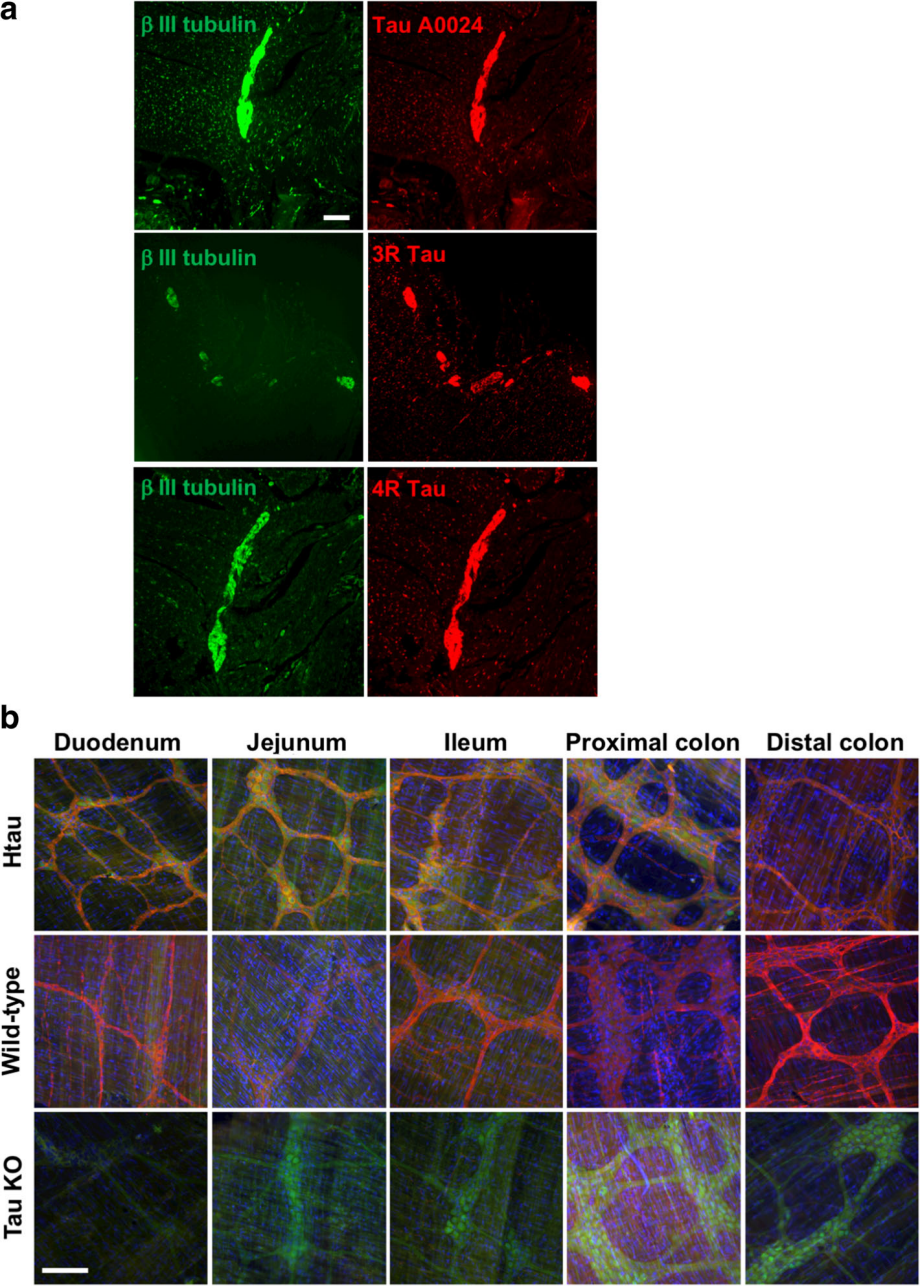
Distribution and localization of tau in human, htau and wild-type mouse myenteric plexus. **a** Total tau antibody A0024 and the isoform-specific antibodies against 3R and 4R-tau were used to detect tau in the myenteric plexus of a human colonic sample. An antibody specific to betaIII-tubulin was used to specifically label neurons. Scale bar is 200 μm (**b**) Total tau antibody A0024 was used to detect tau in the myenteric plexus of the duodenum, jejunum, ileum, proximal colon and distal colon of 2-month-old htau, wild-type (WT) and tau knockout (KO) mice. Merged images show tau (red), EGFP (green) and Hoechst 33342 labelling of nucleic acids (blue). Scale bar is 100 μm. Images are representative of three independent experiments

Immunohistochemistry was used to examine the localization of tau proteins in the human and rodent ENS. Human colonic myenteric plexus showed intense tau immunoreactivity in both neuronal cell bodies and processes when pan-tau A0024, 3R and 4R antibodies were used, which nearly completely overlapped with beta-tubulin immunostaining (Fig. 4a).

In order to examine the localization of tau proteins in the ENS of htau and wild-type mice, sections of small intestine (duodenum, jejunum, ileum) and large intestine (proximal colon and distal colon) were dissected from 2-month-old htau, wild-type and tau knockout mice to isolate the myenteric plexus. Tissue was immunolabelled with an antibody against total tau (A0024). eGFP fluorescence was also imaged as it is inserted in tau exon 1 to disrupt tau expression in tau knockout and htau mice [3]. Tau proteins were found to be abundant throughout the GI tract of htau and wild-type mice, including in the duodenum, jejunum, ileum, proximal colon and distal colon (Fig. 4b). There were no apparent differences in neuronal tau localisation between these regions. Htau proximal colon exhibited dense ganglia and axons and a robust tau signal, whereas the axons and ganglia in htau ileum and WT jejunum were less dense and the resulting tau signal was comparatively less intense. Tau KO express GFP which is observed, and show no tau immunoreactivity. Thus, tau protein is expressed in the myenteric plexus throughout the GI tract of wild-type and htau mice.

### Tau isoforms are phosphorylated in mature ENS but are not susceptible to dephosphorylation with lambda phosphatase

The phosphorylation of tau at multiple serine and threonine sites has been described in both developing and adult brain and is the predominant mechanism by which tau functions are regulated [32]. This logically led us to analyze tau phosphorylation in mature human ENS. Two antibodies specific for tau phosphorylated at Ser202/Thr205 (AT8) [26] and Ser396 (PHF13) [19] detected one single band at 53–54 kDa in colon surgical specimen and biopsies (Fig. 1b and c), thereby demonstrating that the enteric 1N3R and 0N4R tau isoforms are phosphorylated on serine residues under physiological conditions.

**Fig. 5:**
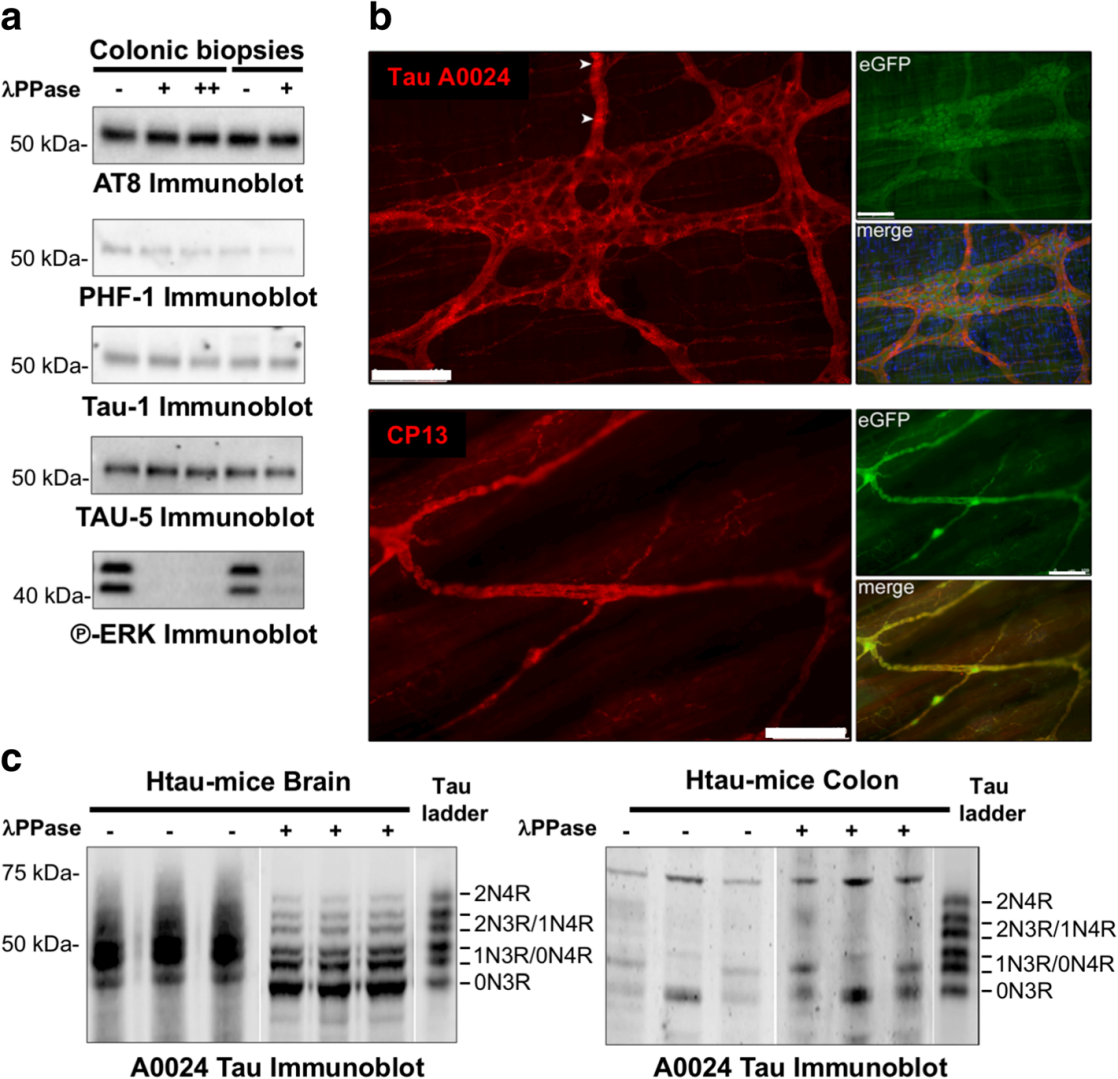
Poor susceptibility of tau to dephosphorylation in mature ENS. **a** Sigmoid colon biopsies lysates from 2 control subjects (#183, 3 first lanes and #208, 2 last lanes) were subjected to immunoblot analysis using TAU-5, AT8, PHF-1 and Tau-1 and antibodies. Lysates were treated with (+ for 1 h and ++ for 3 h) or without (−) lambda phosphatase before immunoblotting. The effectiveness of dephosphorylation was confirmed by phospho-ERK immunoblot (P-ERK immunoblot). **b** Antibodies against total tau (Tau antibody A0024) and tau phosphorylated at serine 202 (CP13) (both shown in red) were used to detect tau in the myenteric plexus of the proximal colon from htau mice. eGFP expression is shown in green, together with merged images including Hoechst 33,342 nuclear labelling (blue). Scale bars are 100 μm. *N* = 3. **c**. Brain and proximal colon homogenates from htau mice were treated with or without lambda phosphatase (+ or -) and immunoblotted with pan-Tau antibody A0024. A recombinant human tau ladder was included on each blot. White lines indicate rearrangement of lanes within the same blot for clarity. Data and images are representative of three independent experiments

We were nevertheless struck by the fact that, in contrast to the brain, lambda phosphatase treatment did not appear to influence the charge/mobility of tau bands in human colon samples when the pan-Tau antibody A0024 was used (Fig. 1a). To further investigate if tau can be dephosphorylated in adult human ENS, colonic biopsy lysates were treated with lambda phosphatase and western blots of these samples were probed with the phospho tau-specific antibodies AT8 (phospho-Ser202/Thr205), PHF1 (phospho-Ser396/404) and Tau-1 (dephospho-Ser199/202/Thr205). Antibodies against ERK were used to check the efficiency of treatment. Although lambda phosphatase efficiently dephosphoylated ERK, it did not modify the phosphorylation state of tau, suggesting that tau is relatively resistant to dephosphorylation in the human adult ENS (Fig. 5a). To further examine tau phosphorylation in mature ENS, we analyzed enteric tau phosphorylation and dephosphorylation in htau mice. Tau is phosphorylated in htau mouse ENS, as phospho-Ser202-positive tau was detected in their colonic myenteric plexus (Fig. 5b). Samples of brain and proximal colon from 2-month-old htau mice were immunoblotted with the pan-Tau antibody A0024. Htau brain showed prominent tau bands ranging from 45 to 70 kDa, in agreement with previous reports [3, 52]. Treatment of these samples with lambda phosphatase to dephosphorylate tau showed that all six major isoforms of tau are expressed in htau brain; these showed good alignment with a recombinant human tau ladder (Fig. 5c). Multiple tau immunoreactive bands ranging from approximately 25–70 kDa were detected in samples from htau proximal colon (Fig. 5c). Two doublets of bands at approximately 45-50 kDa and 55–60 kDa were apparent, which likely corresponds to full-length tau with 0 or 1 N terminal inserts, likely the 1N3R and ON4R isoforms. Moreover, and in contrast to results with htau brain samples, lambda phosphatase treatment did not appear to influence the charge/mobility of tau bands in proximal colon samples (Fig. 5c).

### Tau expression levels are unaltered in the ENS in PSP

**Fig. 6:**
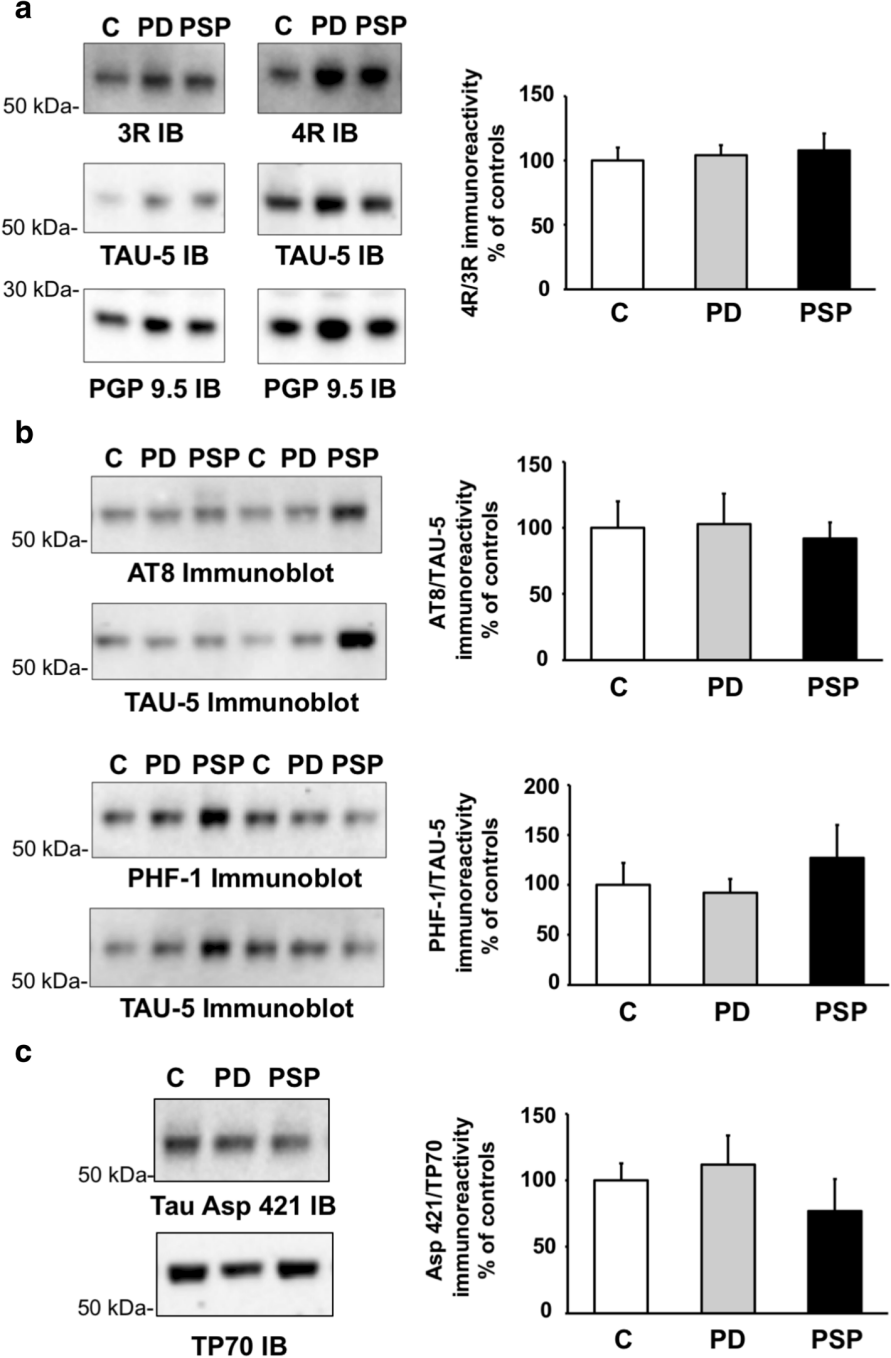
Tau expression and post-translational modifications in colonic biopsies from patients progressive supranuclear palsy, Parkinson’s disease and control subjects. **a** Biopsies lysates were subjected to immunoblot analysis using antibodies against total tau (TAU-5) and against 3R and 4R isoforms. An antibody against protein gene product (PGP) 9.5 was used as a loading control. For quantification, the optical densities of tau-immunoreactive bands were measured, normalized to the optical densities of PGP9.5 immunoreactive bands in the same samples and expressed as percentages of controls. **b** Biopsies lysates were subjected to immunoblot analysis using AT8, PHF-1 and TAU-5 antibodies. The optical densities of phospho-tau-immunoreactive bands were measured, normalized to the optical densities of TAU-5 immunoreactive bands in the same samples, expressed as percentages of controls. **c** Biopsies lysates were subjected to immunoblot analysis using antibodies Tau Asp421 and TP70. The optical densities of immunoreactive bands were measured, normalized to the optical densities of TP70 immunoreactive bands in the same samples, expressed as percentages of controls. Data correspond to mean ± SEM for 9 control samples (C), 10 from Parkinson’s disease (PD) patients and 5 from progressive supranuclear palsy (PSP). Immunoblot (IB)

An increase of the 4R tau to 3R tau isoform ratio has been described in some brain regions in PSP [38]. We thus analyzed the expression levels of tau and the relative abundance of 3R and 4R isoforms in the ENS in colonic biopsies from 5 PSP patients in comparison to colonic samples from 10 PD patients and 9 controls devoid of neurodegenerative disorders. Clinical features of the study population are shown in Table 1. The expression levels of total tau as assessed by immunoblots using the Tau-5 antibody, and the 3R/4R ratio was found not to differ between PSP samples and those from PD and controls (Fig. 6a).

### Tau phosphorylation and truncation in the ENS are similar in PSP, PD and control subjects

Abnormal phosphorylation of tau is a characteristic feature of PSP brain [24, 49] and we therefore analyzed the phosphorylation state of tau in colonic biopsies from PSP patients using the AT8 and PHF-1 antibodies. There were no apparent alterations in tau phosphorylation at these sites in PSP samples in comparison to those from PD and controls, or between PD and controls (Fig. 6b).

Besides abnormal phosphorylation, tau is also truncated in the pathological deposits observed in tauopathies, and especially in PSP [31, 51]. C-terminal tau truncation by caspase-3 was evaluated using a Tau Asp421 antibody, which is specific for tau cleaved at Asp421, along with an antibody against the extreme C-terminus of tau (TP70) [59]. Quantification of the immunoreactive bands detected by Tau Asp421 and TP70 showed no difference in tau truncation at Asp421 and the presence of an intact C-terminus, between PD, PSP and control subjects (Fig. 6c).

### Four tau isoforms are expressed and phosphorylated in primary culture of rat ENS

**Fig. 7:**
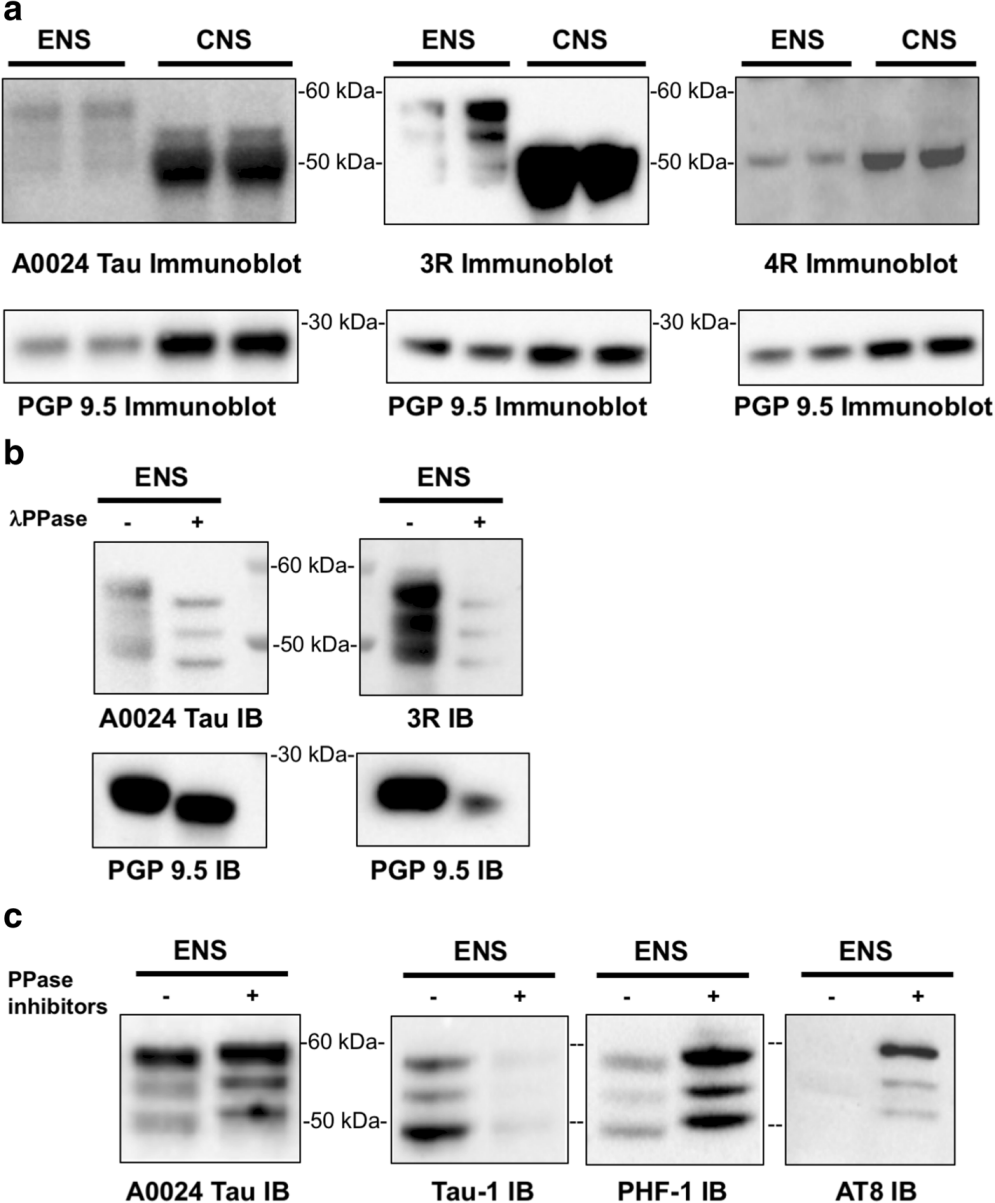
Tau isoform profile and phosphorylation state in rat primary culture of ENS. **a** Lysates of rat primary ENS and CNS cultures were subjected to immunoblot analysis using the pan-Tau antibody A0024 and the isoform specific antibodies 3R and 4R. **b** Primary culture lysates were treated with (+) or without (−) lambda phosphatase before immunoblot analysis with the pan-Tau antibody A0024 and the isoform specific antibody against 3R-tau. PGP9.5 was used as a loading control. **c** Primary culture of rat ENS were treated (+) or not (−) with a cocktail of 3 phosphatase inhibitors including 1 μM okadaic acid, 1 μM ciclosporine A and 6.75 μM sanguinarine (Ppase inhibitors) for 1 h. Fifteen μg of cell lysates were subjected to immunoblot analysis using Tau-1, AT8 and PHF-1 antibodies. IB is for immunoblot. The results shown in (**a**), (**b**) and (**c**) are representative of 2, 4 and 3 independent experiments, respectively

Primary neuronal cultures of rat CNS neurons, which primarily express the shortest tau isoforms 0N3R and 0N4R, have been widely used for studying tau expression, aggregation and secretion [13, 52, 56]. The brain is not the only source from which neurons can be cultured and there are now established protocols for the isolation of enteric neurons from rodents and especially rats. These have already been shown to be useful for studying the expression of neuronal proteins involved in neurodegeneration such as alpha-synuclein [54], however the expression pattern of tau isoforms in rat primary ENS culture remains to be determined. As a first approach to identify tau isoforms in cultured rat enteric neurons, we compared the banding pattern on western blots of total tau as evaluated with the A0024 pan-Tau antibody between primary culture of ENS and cortical neurons. In keeping with previous observations [13, 52], this antibody detected a tau doublet with one major band at 50 kDa and a fainter one around 53 kDa in CNS neurons, which likely correspond to 0N3R and 0N4R isoforms, respectively (Fig. 7a). In ENS neurons, the observed banding pattern was markedly different with a triplet of 50, 53 and 58 kDa bands observed, the latter showing the most intense labelling (Fig. 7a). Further blotting with the 3R and 4R specific antibodies identified 0N3R, 1N3R/0N4R and 2N3R as the main component of the tau triplet observed in primary culture of ENS, while 0N3R and 0N4R were the two primary tau isoforms expressed by primary culture of CNS (Fig. 7a).

Phosphorylation of tau at multiple serine and threonine sites can be modulated in primary culture of CNS [13]. To determine whether tau phosphorylation can be also regulated in primary culture of rat ENS, we treated the cells with either lambda phosphatase or a combination of serine/threonine phosphatase inhibitors. Treatment with lambda phosphatase caused tau dephosphorylation, as evidenced by a significant downward shift in mobility of the tau triplet detected with either the pan-Tau A0024 or 3R antibodies (Fig. 7b). Conversely, treatment with phosphatase inhibitors induced tau phosphorylation as shown by upward shift in mobility of the protein on Western blots probed with the pan-Tau A0024 antibody, and the disappearance of all immunoreactive bands when the Tau-1 antibody against dephosphorylated tau was used (Fig. 7c). When the AT8 antibody was used, no signal was observed under basal conditions, while 3 immunoreactive bands were detected in the presence of phosphatase inhibitors (Fig. 7c). The PHF-1 antibody also detected three immunoreactive bands in untreated cells. An increase in signal intensity along with a mobility shift of all 3 bands was observed following treatment of primary ENS cultures with phosphatases inhibitors (Fig. 7c). Thus, the phosphorylation of ENS tau can be modified, at least in an in vitro setting.

### 3R and 4R tau are differentially expressed in rat primary enteric neuron cultures

**Fig. 8:**
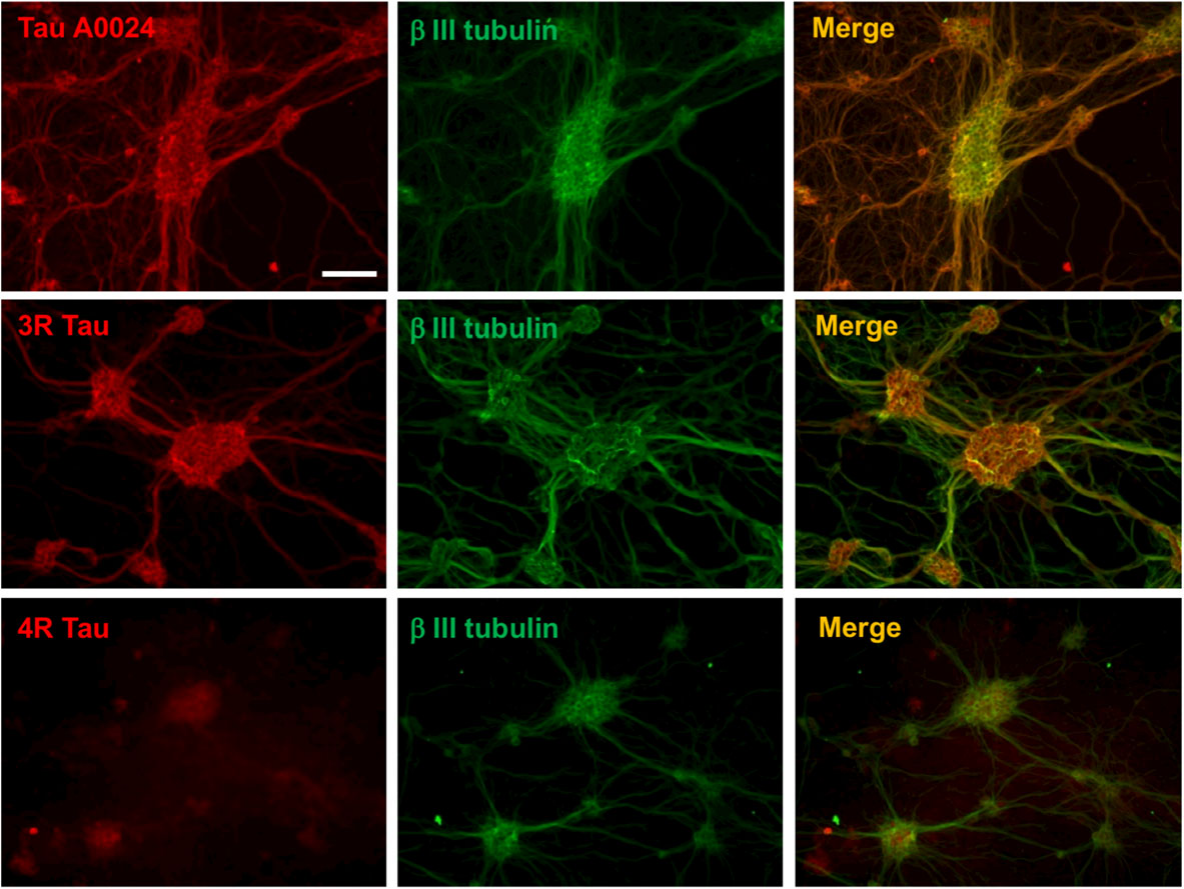
Distribution and localization of tau in primary culture of rat ENS. After 14 days in culture, primary culture of rat ENS were immunostained with the pan-Tau antibody A0024 and the isoforms specific antibodies against 3R and 4R-tau. Scale bar is 100 μM

Lastly, the distribution of tau in rat enteric neurons in culture was examined by immunohistochemistry using pan-Tau A0024, 3R and 4R-tau antibodies at 14 days in vitro. Total tau immunoreactivity was observed in both soma and neuronal processes and the staining patterns produced by pan-Tau A0024, 3R-tau and beta III tubulin antibodies were virtually superimposable (Fig. 8). The 4R-tau staining pattern was markedly different from that observed with 3R-tau and was primarily limited to the cell bodies (Fig. 8). These data indicate that 3R and 4R tau species have different localization in rat primary ENS neurons.

## Discussion

Here, we have used samples of brain and gut from humans, htau transgenic mice and rat primary cultures to show that the isoform profile of tau differs between the ENS and the CNS. We identified 1N3R and 0N4R as the two main tau isoforms expressed in adult human ENS and observed an apparent difference in the relative abundance of different tau isoforms in htau gut and brain, with 1 N and 2 N tau isoforms being over-represented at mRNA levels in htau gut tissues, although 0 N and 1 N isoforms were the predominant protein species detected. We also found that primary culture of rat ENS express four isoforms of tau contrasting with the predominant expression of the single fetal tau 0N3R isoform in rat primary cortical neurons. The ENS and the CNS both contain integrated nervous networks and the similarities between them, including between neurons and glia at a morphological level, have led to the ENS being described as the ‘brain in the gut’ or the ‘second brain’ [22]. Our current and previous results suggest that this anatomical resemblance does not extend to the molecular level as the ENS expresses only a limited number of isoforms of neuronal and glial markers as compared to the brain [10], although the functional consequences of these differences still remain to be determined.

Tau was found to be expressed in both the myenteric and submucosal plexus of human colon and throughout the ENS of wild-type mice and in the htau mouse model. In both the human and rodent ENS, tau protein had a mainly axonal and somatic distribution, which might be expected since in physiological conditions, tau is described as being a predominantly axonal protein [32]. The presence of nuclear tau has been documented in a wide variety of cell and animal systems, including human and rodent brains and neuronal cell lines (reviewed in [32]). So far, the transcript encoding nuclear tau has not been formally identified but findings obtained in mouse brain suggest that the 1N4R isoform is preferentially localised in the nucleus [46]. Although our immunofluorescence experiments performed in mature human and mouse ENS, as well as in primary culture of rat ENS, clearly showed that tau was mainly axonal and somatic, we cannot rule out that a small proportion of enteric tau could also be nuclear. Further experiments including high resolution imaging and biochemical subcellular fractionation will be needed to answer this question.

A panel of well-characterised phospho-specific tau antibodies were used to show that tau is phosphorylated in the ENS of healthy subjects at Ser202/Thr205 and Ser396/Ser404. Tau is known to be phosphorylated at these sites under physiological conditions, with elevated phosphorylation at these epitopes chacteristic of pathological conditions in the CNS (reviewed in [53]). There is mounting evidence to suggest that tau phosphorylation plays a key role in neuronal physiology. The function of tau is strongly affected by its phosphorylation status, influencing its ability to interact with signaling proteins and kinases [57], its association with microtubules and membranes and its ability to regulate axonal transport [58]. Phosphorylation of Ser202/Thr205 and Ser396/Ser404 is commonly found in primary cortical neurons under basal conditions [2] as well as in snap-frozen brain biopsies from subject devoid of neurodegenerative conditions [47], suggesting that these sites are involved in the normal physiology of the CNS. This is further reinforced by the recent observation showing the presence of endogenous tau phosphorylated at these sites at postsynaptic sites in hippocampal neurons where tau interacts with the PSD95-NMDA receptor complex to regulate synaptic activity [48]. These results obtained in the CNS could be extended to the ENS where neuronal plasticity has also been described following modulation of neuronal activity [9, 37].

Soluble tau from adult human brain consist of a heterogeneous mixture of tau isoforms in multiple states of phosphorylation [25, 28]. Because normal electrophoresis techniques do not separate the individual tau isoforms, correct identification of the isoform composition of soluble tau requires an efficient dephosphorylation reaction with lambda phosphatase before immunoblotting [35]. Dephosphorylation of tau from normal adult human brain classically produces a downwards shift enabling a more precise separation and identification of the six tau isoforms [25, 35]. We therefore used the same approach in mature human ENS and the gut of htau mice. In sharp contrast to results with human and htau brain samples, lambda phosphatase treatment did not change the charge/mobility of tau bands in colon samples, suggesting that gut tau may have not been efficiently dephosphorylated. Since western blot and immunohistochemical findings showed that tau in gut is in fact phosphorylated, at least at Ser202, Thr205 and Ser396/ Ser404 this raises the possibility that ENS tau is modified in such a way that it is not susceptible to dephosphorylation. This relative resistance to dephosphorylation, which might be due to conformational changes occurring in case of phosphorylation at some specific sites [18], is specific to adult ENS tau as lambda phosphatase efficiently dephosphorylated tau in rat primary ENS cultures prepared from fetal rats.

In 1978 the first study on cultured myenteric neurons was published [40] and since then there has been a growing interest in this method with several different culture preparations being developed. Using primary cultures of rat ENS [9, 11], we have shown that fetal rat enteric neurons express four isoforms of tau, including the three 3R isoforms. This again stands in sharp contrast to the CNS as rat primary cortical neurons primarily express the 0N3R isoform (our study and [13]). We also show that tau isoforms present in primary ENS culture are phosphorylated under basal conditions and their levels of phosphorylation can be down or upregulated. This suggests that cultured ENS might be helpful to study the regulation of tau expression, phosphorylation and secretion not only in physiological conditions but also in the context of enteric neuropathies [55].

We did not observe any pathological tau changes in the ENS of PSP patients. This stands in sharp contrast with the fact that PSP is considered a prototypical tauopathy of the CNS characterized by tau hyperphosphorylation and truncation [31, 67] and an imbalance in 4R/3R ratio [38]. We have recently proposed that the ENS may be a mirror on to the PD pathology of the CNS since it recapitulates several of the neuronal and glial changes observed in the brain [10, 15, 45]. Our results suggest that, unlike PD, the pathological process in PSP is limited to the CNS and does not involve the ENS. This is supported by the paucity of studies reporting that the peripheral nervous systems are affected in PSP (reviewed in [64]) and by our observation of a lack of glial reaction in the gut in PSP patients [10]. One obvious limitation of this work is that our analysis of PSP samples was restricted to the analysis of the submucosal plexus. We can therefore not rule out that the absence of overt pathological changes in tau in colonic samples from our PSP patients may be due to this limited regional analysis and perhaps different findings would have been obtained had we examined the myenteric plexus. The refinement of new endoscopic procedures, such as full thickness biopsies [50], which provide access to both myenteric and submucosal plexi, may help to answer these critical questions. A second limitation in our study is the lack of neuropathological confirmation of PD and PSP, as the clinical diagnosis of both disorders may have a relatively poor accuracy [1, 42], especially for PD patients for whom signs and symptoms have been present for less than 5 years [1]. In addition, we can not rule out that some of our control subjects may have asymptomatic tauopathy [12].

## Conclusions

We have characterised tau in the human and rodent ENS under physiological conditions and tauopathies. We show differences in tau isoform expression at mRNA and protein level, and in the susceptibility of tau to be dephosphorylated in the CNS and ENS. The data we have acquired on tau in the ENS strongly supports additional future studies aimed at expanding our knowledge of peripheral pathology in neurodegenrative disorders of the CNS and in enteric neuropathies [14].

## Additional file

Additional file 1: **Figure S1.** Validation of the Cosmo-bio 4R antibody. (PDF 220 kb)

## Abbreviations

AD: Alzheimer’s disease
BSA: Bovine serum albumen
CNS: Central nervous system
ENS: Enteric nervous system
ERK: Extracellular signal-regulated kinases
GI: Gastrointestinal
KO: Knockout
MP: Myenteric plexus
PCR: Polymerase chain reaction
PD: Parkinson’s disease
PSP: Progressive supranuclear palsy
Ser: Serine
SMP: Submucosal plexus
TBS: Tris-buffered saline
Thr: Threonine
