# Supplementary material for: Characterisation of tau in the human and rodent enteric nervous system under physiological conditions and in tauopathy
Source: Acta Neuropathol Commun. 2018 Jul 23;6:65. doi: 10.1186/s40478-018-0568-3 (PMC6055332; doi:10.1186/s40478-018-0568-3)
Supplement: Supplementary file 1 — Figure S1. Validation of the Cosmo-bio 4R antibody. (PDF 220 kb) [file 40478_2018_568_MOESM1_ESM.pdf]

Supplementary Figure 1

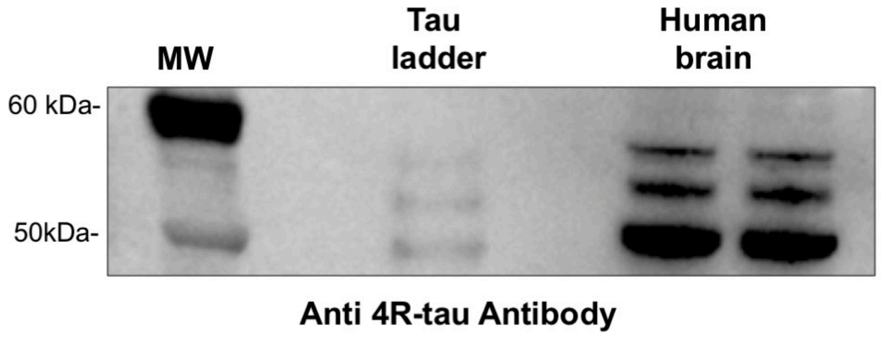

Supplementary figure 1. Validation of the Cosmo-bio 4R antibody. Brain lysates and tau ladder were subjected to immunoblot analysis using the anti 4R-tau antibody from Cosmo-Bio (Ref # CAC-TIP-4RT-P01). This antibody detects 3 specific bands in human brain lysates and in tau ladder.
